# Editorial Note: Incidence and predictors of HIV related opportunistic infections after initiation of highly active antiretroviral therapy at Ayder Referral Hospital, Mekelle, Ethiopia: A retrospective single centered cohort study

**DOI:** 10.1371/journal.pone.0332767

**Published:** 2025-09-22

**Authors:** The PLOS One Editors

The *PLOS One* Editors issue this Editorial Note to inform readers that PLOS has concerns about the article’s [1] authorship and that the peer review did not meet the expected standards for *PLOS One*.

We regret that the issues were not identified and addressed prior to the article’s publication.

This note is not intended to question the quality of the article or contributions of the authors to the review process.
